# Training can’t always lead to Olympic macrophages

**DOI:** 10.1172/JCI158468

**Published:** 2022-04-01

**Authors:** Erwan Pernet, Renaud Prevel, Maziar Divangahi

**Affiliations:** 1 Meakins-Christie Laboratories, Department of Medicine, Department of Microbiology and Immunology, Department of Pathology, McGill University Health Centre, Montreal, Quebec, Canada.; 2 McGill International TB Centre, Montreal, Quebec, Canada.

## Abstract

Although the memory capacity of innate immune cells, termed trained immunity (TI), is a conserved evolutionary trait, the cellular and molecular mechanisms involved are incompletely understood. One fundamental question is whether the induction of TI generates a homogeneous or heterogeneous population of trained cells. In this issue of the *JCI*, Zhang, Moorlag, and colleagues tackle this question by combining an in vitro model system of TI with single-cell RNA sequencing. The induction of TI in human monocytes resulted in three populations with distinct transcriptomic profiles. Interestingly, the presence of lymphocytes in the microenvironment of monocytes substantially impacted TI. The authors also identified a similar population of monocytes in various human diseases or in individuals vaccinated with bacillus Calmette-Guérin. These insights warrant in-depth analysis of TI in responsive versus nonresponsive immune cells and suggest that modulating TI may provide a strategy for treating infections and inflammatory diseases.

## Trained immunity

The dogma that only adaptive immune cells are able to generate immune memory has been challenged by studies in simple organisms (e.g., plants or invertebrates), as well as complex organisms (e.g., vertebrates), defining the existence of memory in innate immune cells (1). Trained immunity (TI) is induced following the exposure to specific training agents such as live bacteria (e.g., bacillus Calmette-Guérin [BCG]) or pathogen-associated molecular patterns (PAMPs; e.g., β-glucan) that epigenetically change the functional immune status. Notably, following removal of the initial training agents, the cellular immune activation returns to basal levels. However, trained immune cells mount faster and enhanced responses to a secondary homologous or heterologous stimulus due to the initial epigenetic imprinting (1). The classical TI response is characterized by increased secretion of proinflammatory cytokines (such as TNF-α, IL-6, and IL-1β; ref. 2) and enhanced antimicrobial capacity (3, 4) or antitumor activity (5) compared with untrained cells. For these reasons, TI has emerged as a promising therapeutic strategy and has been the subject of extensive research.

The discovery of TI in humans was first established in blood monocytes differentiating into macrophages (2). Theoretically, however, TI can be induced in every immune and nonimmune cell type (6). Therefore, the location of this event has been divided into central training in the bone marrow, which is home to hematopoietic stem and progenitor cells (HSPCs) (4, 7, 8), and peripheral training in blood circulation or stromal and structural cells. Although some mechanisms for epigenetic remodeling and metabolic reprogramming of trained human monocytes have been studied (9), the heterogeneity, duration, and maintenance of chromatin modifications driving innate memory responses are still under investigation. In this issue of the *JCI*, Zhang, Moorlag, et al. elegantly investigated the effect of various training agents on the induction of TI in human monocytes/macrophages at single-cell resolution. Additionally, the authors showed the potential contribution of adaptive immune cells to the magnitude of induced TI. Finally, they validated their findings using recently published data sets in monocytes/macrophages isolated from the blood of patients with various illnesses (e.g., ulcerative colitis, sepsis, and COVID-19) or BCG-vaccinated individuals (10).

## Heterogeneity of trained cell populations

**Figure 1:**
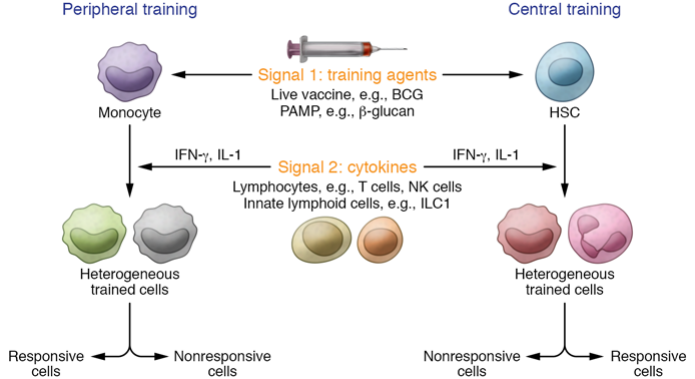
Central and peripheral heterogeneous TI. The induction of peripheral (e.g., monocytes) or central (e.g., hematopoietic stem cells, HSCs) TI involves integration of multiple signaling waves. Stimulation with a training agent (BCG, β-glucan) initiates the first signal (signal 1). The training program of monocytes and HSCs is further potentiated by the second signal (signal 2), which includes cytokine signaling (IFN-γ, IL-1), secreted by lymphocytes (T cells an NK cells) or innate lymphoid cells. Zhang, Moorlag, et al. (10) assessed the transcriptomic profile of peripheral training and identified heterogeneity (responsive vs. nonresponsive cells) in TI of human monocyte/macrophage populations.

To better understand the specific signatures of TI induction in human monocytes and macrophages, Zhang, Moorlag, et al. used four different training agents: β-glucan and muramyl dipeptide to mimic microbially mediated training, and uric acid and oxidized LDL to mimic sterile inflammation–mediated training (2, 3, 11–13) (Figure 1). Using single-cell SORT-seq, they first analyzed the transcriptomic profiles of monocytes after four hours of stimulation with these training agents. Focusing on TI-induced signature genes, i.e., proinflammatory cytokines (TNF-α, IL-6, and IL-1β) and chemokines (CXCL9–11), the authors observed that despite different levels of induction of TI-associated transcriptional programs across the four stimuli, β-glucan was the strongest inducer of TI in monocytes (10). After 5 days of culture, the training agents had no impact on monocyte differentiation into two distinct macrophage populations. Next, the authors assessed the response of trained macrophages upon secondary stimulation (with lipopolysaccharide [LPS]). By using unsupervised cluster analysis, they identified three distinct subsets of macrophages, equally present across the different stimuli. Two of these subpopulations were responsive, with the TI transcriptomic signature indicating high levels of gene expression for proinflammatory cytokines and/or chemokines. Surprisingly, the third subpopulation (38% of the macrophages) was nonresponsive and showed no TI signature (10). Following these in vitro observations, the authors validated their findings using recently published data sets from various human diseases. Both responsive and nonresponsive monocyte/macrophage subsets were present and associated with disease severity.

These exciting and unexpected observations raise several important questions. Although the authors elegantly described the heterogeneity of the monocyte-derived macrophage populations after training, the mechanism that dictates the commitment of cells toward the two distinct responsive and nonresponsive cell types is still unknown. It is intriguing that a substantial fraction of the macrophages remained untrained. The authors speculated that the induction of TI is a dynamic process integrating multiple signaling pathways. The alteration of the cytokine milieu by the responsive cells can trigger both autocrine and paracrine signals to induce or inhibit TI in bystander cells. For example, a first wave of training can induce the expression of protraining cytokines, such as IFN-γ by NK cells or T cells (4, 14), followed by inhibitory signaling pathways such as SHIP1 (15). Consequently, within the same population, there are specific epigenetic and metabolic programs that can promote or limit TI. In addition, our knowledge of cell plasticity for epigenetic imprinting and the dynamic of epigenetic alteration in progenitor versus fully differentiated cells is still limited. Thus, fully differentiated macrophages might lose their plasticity for gaining new epigenetic changes following stimulation. Therefore, coupling the transcriptomic landscape with single-cell analysis of accessible chromatin (ATAC-seq) of trained immune cells will be a powerful approach to address these questions.

While these nonresponsive cells limit the overall magnitude of the host response, we can speculate that they may represent an evolutionary mechanism to regulate monocyte and macrophage activation and prevent induction of maladaptive responses. Interestingly, the authors have demonstrated that TI signatures were suppressed in monocytes from patients with severe sepsis or COVID-19. Therefore, systematic functional assessment of responsive versus nonresponsive innate immune cell populations is required to understand the full spectrum of trained immune cells in the setting of infectious or immune-mediated diseases.

## Role of lymphocytes in TI

In parallel to studying the heterogeneity of the trained monocyte and macrophage populations, Zhang, Moorlag, et al. also tackled the potential crosstalk between innate and adaptive immune cells in augmenting TI. They used a simple but efficient approach by comparing the training capacity of monocytes in total PBMCs versus Percoll-isolated monocytes (10). Using this approach, the authors observed that the presence of other leukocytes in the microenvironment of monocytes enhanced their transcriptional response to the training agents. This observation indicates that the presence of other cells can have a profound impact on the commitment of monocytes toward trained phenotypes. Using NicheNet to analyze cell-cell interactions inferred from their single-cell RNA-seq data set, the authors suggested that monocytes actively communicate with lymphocytes. In particular, signals from NK cells and CD8^+^ T cells potently amplified TI. In line with this observation of peripheral training, studies of central training show that the HSPC training program requires type II IFN (IFN-γ) signaling after BCG vaccination (4, 14) or IL-1 signaling after β-glucan training (3). Interestingly, it is important to note that virulent pathogens (e.g., *Mycobacterium* *tuberculosis*) are able to use the type I IFN signaling pathway to inhibit TI (7). Collectively, these studies indicate that identifying the cellular source of these key cytokines and the magnitude of pro- and anti-training dialogue between adaptive and innate immune cells will be required to determine the complex mechanisms involved in TI.

## A role for eicosanoids in TI?

Remarkably, through their analysis of in vitro and in vivo data sets, Zhang, Moorlag, and colleagues identified additional TI signature genes (10), including *PTGS2*, which encodes cyclooxygenase 2 (COX2) that is required for the generation of bioactive lipids such as prostaglandin E2 (PGE2). This result agrees with a previous study demonstrating that NK cell–derived IFN-γ induces a regulatory program in monocytes, including increased PGE2 production, prior to egress from the bone marrow (14). However, whether eicosanoids are required for inducing TI or the alteration of eicosanoids is a consequence of TI remains to be elucidated. Nonetheless, because PGE2 and other eicosanoids are master regulators of the host resistance and disease tolerance to both bacterial and viral infection (16–20), they may also regulate the magnitude of TI peripherally or centrally. A careful lipidomic analysis of trained immune cells will be required to identify the effect of TI on the global eicosanoid profile of monocytes and macrophages. Thus, modulation of TI by using readily available drugs targeting eicosanoid pathways may be a therapeutic avenue in both infectious and noninfectious diseases.

## Conclusions

Considering the multidimensional mechanism(s) of TI, the study by Zhang, Moorlag, et al. provides the first evidence of the heterogeneity (responsive vs. nonresponsive) of TI in human monocyte and macrophage populations and hints at a functional role of these subsets in several human diseases (10). The authors also found that the induction of TI is regulated by other immune cells, in particular lymphocytes, indicating the importance of dialogue between innate and adaptive immune cells for generating and potentially maintaining TI. Undoubtedly, understanding the cellular and molecular networks in TI will provide us tools for developing new therapeutic approaches against complex diseases.

## Version 1 04/01/2022

Electronic publication
